# High impact of COVID-19 in long-term care facilities, suggestion for monitoring in the EU/EEA, May 2020

**DOI:** 10.2807/1560-7917.ES.2020.25.22.2000956

**Published:** 2020-06-04

**Authors:** ECDC Public Health Emergency Team, Kostas Danis, Laure Fonteneau, Scarlett Georges, Côme Daniau, Sibylle Bernard-Stoecklin, Lisa Domegan, Joan O’Donnell, Siri Helene Hauge, Sara Dequeker, Eline Vandael, Johan Van der Heyden, Françoise Renard, Natalia Bustos Sierra, Enrico Ricchizzi, Birgitta Schweickert, Nicole Schmidt, Muna Abu Sin, Tim Eckmanns, José-Artur Paiva, Elke Schneider, ECDC Public Health Emergency Team, ECDC Public Health Emergency Team, Cornelia Adlhoch, Pete Kinross, Angeliki Melidou, Gianfranco Spiteri, Sergio Brusin, Margot Einöder-Moreno, Anastasia Pharris, Senia Rosales-Klintz, Teymur Noori, Orlando Cenciarelli, Diamantis Plachouras, Nick Bundle, Tommi Kärki, Eeva Broberg, Bruno Ciancio, Carl Suetens

**Affiliations:** 1 The members of the ECDC Public Health Emergency Team are listed at the end of the article; 2 Santé Publique France (SpFrance), the French National Public Health Agency, St Maurice, France; 3 European Programme for Intervention Epidemiology Training (EPIET), European Centre for Disease Prevention and Control (ECDC), Stockholm, Sweden; 4 Health Service Executive-Health Protection Surveillance Centre, Dublin, Ireland; 5 Norwegian Institute of Public Health, Oslo, Norway; 6 Sciensano, Brussels, Belgium; 7 Regional Health and Social Agency - Emilia Romagna, Bologna, Italy; 8 Robert Koch-Institute, Berlin, Germany; 9 Directorate General of Health, Lisbon, Portugal; 10 European Agency for Safety and Health at Work, Bilbao, Spain

**Keywords:** COVID-19, long-term care facilities, surveillance, EU/EEA

## Abstract

Residents in long-term care facilities (LTCF) are a vulnerable population group. Coronavirus disease (COVID-19)-related deaths in LTCF residents represent 30–60% of all COVID-19 deaths in many European countries. This situation demands that countries implement local and national testing, infection prevention and control, and monitoring programmes for COVID-19 in LTCF in order to identify clusters early, decrease the spread within and between facilities and reduce the size and severity of outbreaks.

Within a few months the coronavirus disease (COVID-19) spread all over the world. As at 26 May 2020, 5,459,526 COVID-19 cases have been reported globally, of which 1,361,098 (25%) were reported by the European Union/European Economic Area (EU/EEA) and the United Kingdom (UK). Globally, 354,994 cases (6.5%) were fatal, of which 161,063 (45%) occurred in the EU/EEA and the UK [1]. In the EU/EEA and the UK, the majority of hospitalisations and deaths were among the oldest age groups 70 years and older [2]. 

The purpose of this summary is to raise awareness of the severe impact of COVID-19 in LTCF and provide an overview of the importance of surveillance and infection prevention and control (IPC) measures outlined in the guidance documents from the European Centre for Disease Prevention and Control (ECDC) [3,4].

## Long-term care facilities definition

In the context of this work, LTCF were defined to include institutions such as nursing homes, skilled nursing facilities, retirement homes, assisted-living facilities, residential care homes or other facilities that take care of people requiring support who experience difficulties living independently in the community because of the interaction between barriers in the environment and physical, mental, intellectual or sensory impairments possibly related to old age or chronic medical conditions [5]. In 2016 and 2017, the estimated number of nursing homes, residential homes and mixed LTCF in EU/EEA countries was 64,471 with 3,440,071 beds [6].

## Epidemiology of COVID-19 in long-term care facilities

### Risk of introduction and spread 

Residents in long-term care facilities (LTCF) are at high risk for COVID-19 infection and for severe outcomes of COVID-19 as they often belong to older age groups and may be frail, with chronic comorbidities which make recognition of typical COVID-19 symptoms challenging [5]. Circumstances such as insufficient access to personal protective equipment, staff with limited training in IPC, low or absent testing capacity, residents with few or atypical symptoms, asymptomatic staff or staff who work while symptomatic (including with mild symptoms) and staff who work in multiple LTCF can facilitate entry of COVID-19 and occur to varying degrees in LTCF [7]. Two point prevalence surveys in an LTCF in the United States (US) identified spread of COVID-19 in the majority of residents within 23 days after the first case [8]. The prevalence of asymptomatic infections in LTCF remains less clear. Data from Belgium as at 25 May 2020 indicated a high proportion of up to 76% asymptomatic positive-tested residents (1,381 symptomatic; 4,353 asymptomatic) and staff (844 symptomatic; 2,368 asymptomatic) in LTCF. However, a study in the US reported that 24 of 27) of the cases registered as asymptomatic had developed compatible symptoms within 7 days, i.e. they had been pre-symptomatic [8,9]. A broad testing strategy of exposed staff and residents has prevented a facility-wide outbreak through early screening and identification of COVID-19 cases (symptomatic and asymptomatic) in an LTCF in the US [10]. The positive impact of a broad testing strategy on containing outbreaks in these settings has also been reported in another US study where rapid and serial testing was performed [11].

### Situation in the EU/EEA countries 

In several EU/EEA countries, LTCF have been severely affected by COVID-19, with deaths among residents accounting for 37–66% of all COVID-19-related deaths (Table) [12]. As at 25 May, Belgium had one of the highest numbers of reported fatal COVID-19 cases in the EU/EEA, 813 deaths per million inhabitants with 51% possible or confirmed fatal cases reported from LTCF, accounting for 413 deaths per million population [2,9]. 

**Table:** Number of affected facilities (long-term care and other specified settings), COVID-19 cases and deaths among residents, examples from countries in the EU/EEA and the UK, May 2020 (n = 58,831 deaths)

Country	Report date (in 2020)	Affected facilities	Confirmed COVID-19 cases in LTCF residents	COVID-19-related deaths in LTCF residents	Total COVID-19 deaths	% of all COVID-19 deaths in the country	COVID-19 deaths in LTCF/1 million population^a^
Belgium [9]	25 May	Unk	5,734	4,735	9,312	51	413.3
France [16]	29 May	7,923	74,402	14,113	28,530	50	210.6
Germany [17]^a^	25 May	Unk	15,757	3,138	8,257	38	37.8
Ireland [20]^b,c^	25 May	458	6,392	811	1,354	60	165.4
Norway [21]	25 May	Unk	Unk	139	235	59	25.5
The Netherlands [22]	19 May	Unk	9,474	1,779	5,694 [1]	31	102.9
Spain [13]	25 May	5,457	Unk	19,066	28,752 [1]	66	406.2
Stockholm County, Sweden [14]	15 April	212 (400^d^; 53%)	1,711	630	1,400	45	409.6
Sweden [15]	18 May	Unk	2,866	1,777	3,661	49	173.7
UK – England and Wales [23]	15 May	Unk	Unk	11,650	45,226	26	196.0
UK – Scotland [24,25]	17May	655 (60%)	5,652	1,623	3,546	46	297.1

COVID-19: coronavirus disease; EU/EEA: European Union/European Economic Area; LTCF: long-term care facility; UK: United Kingdom; Unk: unknown.

^a^ Eurostat data from 2019 [26].

^b^ Includes homes for the elderly, migrants and homeless as well as prisons.

^c^ Personal communication, Lisa Domegan, 27 May 2020.

^d^ Total number of facilities in Stockholm County provided in the report.

Data for Ireland relate to COVID-19 outbreaks in all residential facilities such as nursing homes for the elderly, centres for those with disabilities, homeless facilities and direct provision centres and for all cases linked to those outbreaks. The data for Ireland include staff and residents.

High incidence (406/1 million inhabitants) among LTCF residents was reported from Spain, where many facilities have been affected, with 17,730 COVID-19-related deaths, representing 66% of all COVID-19 deaths [13]. Also Stockholm county in Sweden has reported similar rates of COVID-19 deaths in LTCF residents (410/1 million population), with a high proportion of affected facilities (51%; 202/400) (Table) [14]. Across Sweden, fatal COVID-19 cases in LTCF residents accounted for 49% of all COVID-19-related deaths by 18 May 2020. Of 8,935 infected staff in these facilities, 46 (0.5%) have died [15]. The fact that around 80,000 of all Swedes being 65 years and older live in a care home underlines the potential impact [15]. Norway, with a far lower overall incidence of cases in the population and just 26 deaths in LTCF residents per 1 million inhabitants, reported that 61% of all fatal cases occurred in LTCF residents. During the same period, France had reported 14,113 deaths in residents, representing 50% of all fatal cases; this also included a large number of staff (> 40,000 cases) [16]. Germany reported that 20% of confirmed infections in residents of special facilities (including LTCF but also others) were fatal, accounting for 38% of all fatal cases [17]. Similar patterns as described above, with high numbers of affected facilities and related deaths, are observed in other countries such as Ireland, the Netherlands or the UK (Table) but comprehensive data from LTCF are not available for many EU/EEA countries [18]. 

Data deriving from outbreak surveillance could cover also cases from other closed settings and therefore underestimate the mortality in residents in LTCF. Although the reported numbers are high and may differ based on case definitions applied, deaths among not laboratory-confirmed probable cases or among people who had not been tested could even increase the numbers and under-reporting of COVID-19-related deaths in LTCF may therefore be assumed.

## ECDC guidance for surveillance and control in long-term care facilities

Few countries in the EU/EEA have surveillance systems specifically for LTCF, although many national monitoring systems record whether infections or outbreaks occurred in an LTCF [19]. Some countries implemented COVID-19-specific surveillance systems, substantially modified existing seasonal influenza surveillance systems, implemented nationwide comprehensive testing for SARS-CoV-2 in LTCF or focussed on monitoring COVID-19-related mortality.

To support EU/EEA countries intending in implementing or revising national surveillance systems, the ECDC published in May 2020 guidance for surveillance of COVID-19 in LTCF, with recommendations for data collection and reporting, accompanied by a data collection tool [4]. The surveillance objectives (Box 1) are to detect COVID-19 cases among LTCF residents and staff early and to limit transmission through IPC.

Box 1 **Proposed surveillance objectives for long-term care facilities**

The objectives of COVID-19 surveillance in LTCF at local, regional and national level as well as EU/EEA level are as follows:

- Detect COVID-19 infections in LTCF residents and staff, to enable appropriate implementation of infection prevention measures, to limit the size of outbreaks (local objective);
- Estimate and monitor the number and proportion of affected LTCF, to provide situational awareness;
- Monitor changes in the transmission and geographical distribution of affected LTCF over time, to assess prevention and control efforts;
- Estimate and monitor the impact and severity of COVID-19 in LTCF, including impact on overall mortality in LTCF residents, in order to provide situational awareness of the current burden of disease and to inform mitigation measures.

COVID-19: coronavirus disease; EU/EEA: European Union/European Economic Area; LTCF: long-term care facility.

LTCF should consider implementing a daily surveillance routine for LTCF residents, to measure fever, respiratory rate, oxygen saturation and other typical and atypical signs and symptoms of COVID-19 infection (Box 2) [4]; they should also consider and strengthen IPC measures [3]. After a possible case (Box 2) is detected in a resident or staff in an LTCF, testing for SARS-CoV-2 should be initiated promptly for the respective person. If a confirmed case is detected, comprehensive testing is recommended for all residents and staff, depending on testing capacity including post-mortem testing. In addition, facilities in an area with ongoing community transmission should test all staff regularly even if there has not been a case inside the facility, if testing is possible. Such regular, e.g. weekly, comprehensive testing of staff will identify asymptomatic and pre-symptomatic cases and those who have unrecognised mild symptoms. Identification of cases should trigger immediate additional IPC and occupational safety and health measures as well as cohorting of infected residents [3]. Infected staff should be dispensed from their duties and quarantined until they test negative in two consecutive samples, collected at least 8 days after the onset of symptoms. Also asymptomatic staff who test positive should be treated as potentially infectious and need to refrain from work for 8 days or at least until they test negative (two negative RT-PCR tests from respiratory specimens at a 24 h interval if testing is available). It is recommended to wear at least a surgical face mask during working hours for 14 days after being tested positive. Any visits to residents in LTCF should be limited to the absolute minimum.

Box 2 **Suggested indicators for the identification of a possible COVID-19 case in a long-term care facility**

Typical symptoms:

- cough, fever, sore throat, shortness of breath, sudden onset of anosmia, ageusia or dysgeusia

Atypical symptoms:

- headache, chills, muscle pain, fatigue, vomiting, diarrhoea

Signs:

- oxygen saturation via pulse oximetry <95%, respiratory rate >25/min

COVID-19: coronavirus disease.

Reporting surveillance data to regional or national public health authorities will provide situational awareness and enable targeted control measures. Frequent reporting to authorities should take place even if there are no cases, on a weekly or monthly basis, to ensure the establishment of lines of communication. If a possible case is detected in an LTCF, weekly reporting may be sufficient in unaffected areas, i.e. those without ongoing community transmission. However, daily reporting is recommended if (i) the LTCF has a sudden increase in possible cases, (ii) the LTCF is in an affected area or (iii) at least one confirmed case is detected among residents or staff. A national comprehensive facility-based electronic reporting system (with the ability to categorise cases as staff or residents) is considered the best option to conduct surveillance in LTCF and fulfil the surveillance objectives.

## Conclusion

COVID-19 affects elderly residents in LTCF considerably, causing high morbidity and mortality. In the absence of vaccination or effective pharmaceutical measures, early identification of virus circulation in LTCF through comprehensive surveillance will help protect LTCF residents and staff. Early testing to identify symptomatic and asymptomatic cases and immediate implementation of additional IPC and occupational safety and health measures as well as cohorting of infected residents will help minimise outbreaks and the overall impact of COVID-19 on the elderly.
